# Inverted U-shaped association between the systemic immune-inflammation index and colorectal polyps in Chinese patients: a cross-sectional study

**DOI:** 10.3389/fmed.2024.1515230

**Published:** 2025-01-27

**Authors:** Jie Bao, Yajie Teng, Yingxue Yang, Qinghua Wang

**Affiliations:** Department of Gastroenterology, First People’s Hospital of Kunshan, Kunshan, China

**Keywords:** systemic immune-inflammation index, colorectal polyps, cross-sectional study, non-linear relationship, China

## Abstract

**Purpose:**

The systemic immune-inflammation index (SII) has been found to be associated with various inflammatory and neoplastic diseases. The aim of this study was to investigate the relationship between the SII and colorectal polyps (CPs) in Chinese patients.

**Methods:**

This was a cross-sectional study. We retrospectively collected data from 3,028 Chinese patients who underwent physical examinations, including colonoscopy, from 2018 to 2022. We conducted a comparative analysis of patient characteristics among those with adenomatous polyps, non-adenomatous polyps, and individuals without CPs using descriptive statistics. We calculated the SII for each group and assessed the relationship between SII values and the presence of CPs.

**Results:**

Our study included 3,028 individuals, of whom 1,432 (47.29%) had colorectal polyps. After adjusting for confounding variables, the natural logarithm of the SII (Ln-SII) was significantly associated with the prevalence of adenomatous polyps in both males and females, with an odds ratio (OR) of 0.76 [95% confidence interval (CI): 0.65–0.88, *p* = 0.0003]. An inverted U-shaped relationship was observed between Ln-SII and the prevalence of colorectal polyps, including both adenomatous and non-adenomatous polyps, with a cut-off point at 5.78 (5.39 for adenomatous polyps and 5.79 for non-adenomatous polyps). Below this cut-off point, a significant association with colorectal polyps was identified, with an OR of 1.73 (95% CI: 1.25–2.40, *p* = 0.0009). Specifically, for adenomatous polyps, the OR was 2.91 (95% CI: 1.03–8.20, *p* = 0.0437), and for non-adenomatous polyps, the OR was 1.86 (95% CI: 1.31–2.65, *p* = 0.0006). Beyond the cut-off point, the association between Ln-SII and colorectal polyps remained significant, with an OR of 0.56 (95% CI: 0.46–0.68, *p* < 0.0001). In the adenomatous polyps group, the OR was 0.57 (95% CI: 0.43–0.74, *p* < 0.0001), and in the non-adenomatous polyps group, the OR was 0.57 (95% CI: 0.46–0.70, *p* < 0.0001).

**Conclusion:**

The inverted U-shaped association between Ln-SII and the risk of colorectal polyps highlights the potential relevance of monitoring variations in SII and suggests that SII may be a promising predictor for colorectal polyp development.

## Introduction

Colorectal polyps (CPs) are abnormal growths of tissue that arise from the mucosal layer of the colon and rectum (1). There are several pathological subtypes, including adenomatous polyps, hyperplastic polyps, inflammatory polyps, and hamartomatous polyps. While many CPs are benign, some have the potential to progress to colorectal cancer (CRC), making their early detection and management crucial for cancer prevention (2). The global incidence of CPs has been steadily increasing, largely attributed to aging populations, dietary changes, and sedentary lifestyles, highlighting the importance of routine screening through colonoscopy (3). In fact, CRC is the third most commonly diagnosed cancer worldwide, with a significant proportion originating from adenomatous polyps (4).

The exact mechanisms that lead to the formation and progression of CPs are complex and multifactorial, involving genetic, environmental, and inflammatory factors (5). Accumulating evidence shows that chronic inflammation and genetic variation play key roles in colorectal carcinogenesis via premalignant polyps (6). The systemic immune-inflammation index (SII) is a novel indicator used to evaluate systemic inflammation, based on peripheral lymphocyte (Lym), neutrophil (Ne), and platelet (Plt) counts. It has gained attention as a reliable predictor of inflammatory states and has been associated with various diseases, including cancers such as CRC (7). Its potential prognostic value has been confirmed in a wide range of conditions (8–10).

Given that early detection and removal of CPs can significantly contribute to the prevention of CRC, our study aimed to investigate the association between the SII and the development of CPs. Specifically, we focused on evaluating the relationship between SII and various polyp subtypes, including adenomatous and non-adenomatous polyps. Understanding these associations may provide insights into early detection strategies and enhance preventative measures for CRC.

## Methods

### Study population

**Figure 1:**
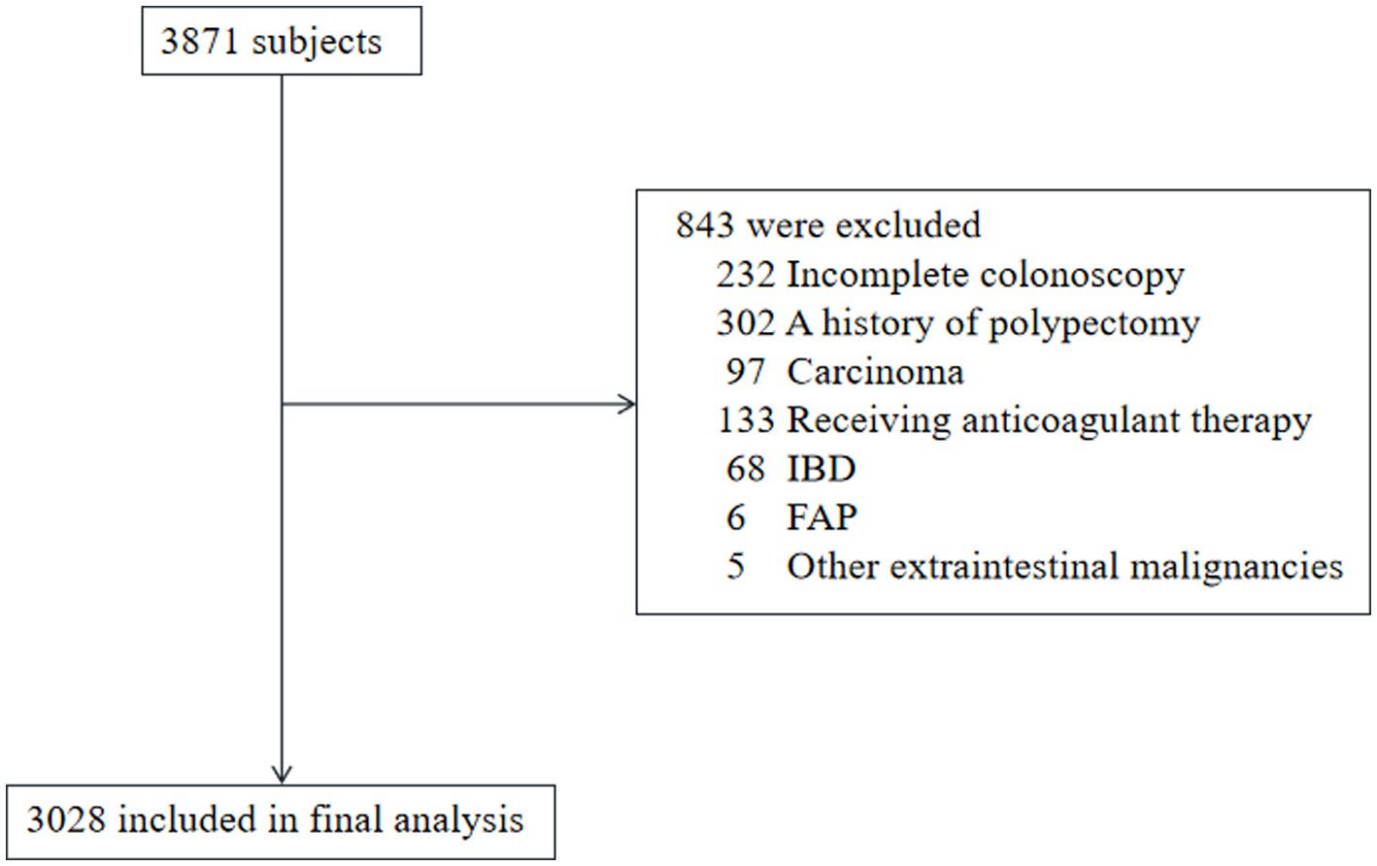
Flow diagram of patient inclusion and exclusion. FAP, familial adenomatous polyposis; IBD, inflammatory bowel disease.

A retrospective analysis was conducted on the medical records of 3,871 adults who underwent routine health evaluations, including blood tests and colonoscopy, at the First People’s Hospital of Kunshan, China, between January 2018 and December 2022. Each participant was assessed using a standardized questionnaire, covering demographic and medical history, alongside a comprehensive physical examination. Ethical approval was obtained from the Ethics Committee of the First People’s Hospital of Kunshan. The requirement for informed consent was waived due to the retrospective nature of the research, utilizing anonymized secondary data. A total of 843 individuals were excluded for reasons such as incomplete colonoscopy, presence of inflammatory bowel disease, prior history of polypectomy or carcinoma, ongoing anticoagulant therapy, familial adenomatous polyposis, or other extracolonic malignancies. A flowchart illustrating the patient selection and exclusion criteria is presented in Figure 1.

### Laboratory examination

For all participants, venous blood samples were drawn from the antecubital vein in the morning following at least 12 h of overnight fasting. Hematological parameters, including white blood cell (WBC) count, lymphocyte (Lym) count, neutrophil (Neu) count, and platelet (Plt) count, were quantified utilizing a Sysmex XN hematology analyzer. Biochemical analyses, including aspartate aminotransferase (AST), alanine aminotransferase (ALT), total cholesterol (TC), triglycerides (TG), high-density lipoprotein cholesterol (HDL-C), low-density lipoprotein cholesterol (LDL-C), uric acid (UA), and glucose (GLU), were conducted through standard enzymatic assays using the VITROS 5600 Integrated System.

### Colonoscopy and pathological examination

All patients underwent bowel preparation prior to colonoscopy using polyethylene glycol electrolyte powder to ensure optimal bowel cleansing. Colonoscopy procedures were performed using the OLYMPUS 290 colonoscope system (Tokyo, Japan) by board-certified gastroenterologists with over 5 years of clinical experience. A successful colonoscopy was defined by advancing the scope to the cecum. Polyps identified during colonoscopy were biopsied or resected for histopathological evaluation performed by experienced pathologists in accordance with WHO classification guidelines. Based on colonoscopic and histological results, patients were categorized into three groups: polyp-free, adenomatous polyps, and non-adenomatous polyps.

### Statistical analysis

Data analyses were performed using R software (version 3.5.3). Continuous variables were expressed as mean ± standard deviation (SD) and compared between groups using the Kruskal–Wallis test. Categorical variables were presented as counts and percentages, and comparisons were made using Fisher’s exact test. Multivariate logistic regression models were applied to identify potential risk factors for adenomatous and non-adenomatous polyps. Variables included in the models were age, sex, and relevant clinical parameters. Odds ratios (ORs) with 95% confidence intervals (CIs) were reported. A *p*-value <0.05 was considered statistically significant. Sensitivity analyses were conducted by stratifying the cohort by sex to validate the consistency of findings.

To further assess the relationship between SII and different pathological types of colorectal polyps, generalized additive models (GAMs) were employed for smooth curve fitting to capture potential non-linear associations.

## Results

### Baseline characteristics

Table 1 outlines the baseline demographic and clinical characteristics of the study population, stratified by colonoscopic findings and pathology. The cohort was categorized into two primary groups: individuals without CPs and those diagnosed with CPs. The latter group was further subdivided into adenomatous and non-adenomatous polyps. Statistical analysis revealed a significant male predominance in the CPs cohort compared to the polyp-free group. Age distribution indicated that individuals with CPs were, on average, older than those without polyps. Hematological parameters, including WBC counts and lymphocyte levels, exhibited significant differences between the groups. Notably, the SII was markedly elevated in patients with CPs, suggesting a potential association with inflammatory processes. Biochemical assessments demonstrated significantly higher levels of liver enzymes, specifically ALT and AST, in the CPs group compared to the polyp-free group. Additionally, lipid profiles indicated elevated TG and TC levels among individuals with CPs. UA levels were also significantly increased in this group. Moreover, FBG levels were higher in those with CPs.

**Table 1:** Demographic and clinical characteristics of subjects according to colonoscopic findings and pathology.

	Polyp-free	Colorectal polyps	*p*-value	Adenomatous polyps	*p*-value	Non-adenomatous polyps	*p*-value
*N* (%)	1,596 (52.71)	1,432 (47.29)		323 (22.56)		1,109 (77.44)	
Male	809	919	<0.001	199	<0.001	720	<0.001
Age (yr)	53.13 ± 15.51	55.85 ± 11.84	<0.001	53.20 ± 12.73	0.980	56.62 ± 11.47	<0.001
WBC (*10^9^/L)	5.75 ± 2.02	5.78 ± 1.59	0.005	5.74 ± 1.55	0.144	5.80 ± 1.61	0.007
NEU (*10^9^/L)	3.66 ± 1.74	3.55 ± 1.36	0.757	3.48 ± 1.33	0.454	3.57 ± 1.37	0.475
LYM (*10^9^/L)	1.60 ± 0.64	1.71 ± 0.55	<0.001	1.74 ± 0.54	<0.001	1.71 ± 0.55	<0.001
PLT (*10^9^/L)	194.88 ± 63.81	198.67 ± 57.22	0.031	198.15 ± 55.76	0.141	198.83 ± 57.67	0.053
SII index	508.83 ± 422.85	453.74 ± 339.95	0.006	434.00 ± 279.97	0.001	459.51 ± 355.50	0.061
ALT (U/L)	27.88 ± 49.06	25.30 ± 25.04	<0.001	27.15 ± 19.75	<0.001	24.78 ± 26.34	<0.001
AST (U/L)	25.59 ± 35.95	22.81 ± 11.58	0.053	23.53 ± 9.45	0.015	22.60 ± 12. 11	0.217
TG (mmol/L)	1.43 ± 1.23	1.64 ± 1.12	<0.001	1.69 ± 1.36	0.001	1.63 ± 1.04	<0.001
TC (mmol/L)	4.26 ± 1.07	4.65 ± 0.97	<0.001	4.57 ± 1.01	<0.001	4.67 ± 0.95	<0.001
UA (mmol/L)	307.91 ± 91.20	330.78 ± 88.62	<0.001	328.63 ± 87.85	<0.001	331.40 ± 88.87	<0.001
FBG (mmol/L)	5.35 ± 1.31	5.47 ± 1.13	<0.001	5.46 ± 1.05	0.002	5.47 ± 1.15	<0.001

WBC, white blood cells; NEU, neutrophils; LYM, lymphocytes; SII, systemic immune-inflammation; ALT, alanine aminotransferase; TG, triglycerides; TC, total cholesterol; UA, uric acid; FBG, fasting blood glucose.

### Ln-SII index is associated with the prevalence of colorectal polyps, particularly adenomatous polyps

As shown in Table 2, our analysis revealed that the logarithmic transformation of the SII (Ln-SII) was inversely associated with the occurrence of CPs, including both adenomatous and non-adenomatous types, when compared to the polyp-free group. In Model I, multinomial logistic regression analysis indicated that a higher Ln-SII index was correlated with a reduced prevalence of CPs (OR = 0.83, 95% CI: 0.73–0.94, *p* = 0.0037). After adjusting for age and sex in Model II, the association remained significant, with an adjusted odds ratio (OR) of 0.81 (95% CI: 0.72–0.92, *p* = 0.0015). In Model III, which adjusted for additional confounding factors, including ALT, AST, TC, TG, UA, and FBG, the inverse relationship strengthened (OR = 0.76, 95% CI: 0.65–0.88, *p* = 0.0003). For adenomatous polyps, the Ln-SII index showed a significant negative correlation across all models. In Model I, the odds ratio was 0.75 (95% CI: 0.61–0.92, *p* = 0.0050), which further decreased to 0.72 (95% CI: 0.59–0.89, *p* = 0.0018) in Model II and to 0.61 (95% CI: 0.48–0.78, *p* = 0.0001) in Model III. In contrast, non-adenomatous polyps also exhibited a significant inverse relationship with the Ln-SII index, with an odds ratio of 0.86 (95%CI: 0.76–0.99, *p* = 0.0328) in Model I, and adjusted odds ratios of 0.84 (95% CI: 0.74–0.97, *p* = 0.0148) and 0.81 (95% CI: 0.69–0.95, *p* = 0.0093) in Models II and III, respectively.

**Table 2:** Adjusted ORs and 95% CIs for the occurrence of colorectal polyps, including adenomatous and non-adenomatous polyps, in relation to the Ln-SII index based on multinomial logistic regression.

Variable	Model I		Model II		Model III	
	OR, 95% CI	*p*-value	OR, 95% CI	*p*-value	OR, 95% CI	*p*-value
Colorectal polyps						
Ln-SII	0.83 (0.73, 0.94)	0.0037	0.81 (0.72, 0.92)	0.0015	0.76 (0.65, 0.88)	0.0003
Adenomatous polyps						
Ln-SII	0.75 (0.61, 0.92)	0.0050	0.72 (0.59, 0.89)	0.0018	0.61 (0.48, 0.78)	0.0001
Non-adenomatous polys						
Ln-SII	0.86 (0.76, 0.99)	0.0328	0.84 (0.74, 0.97)	0.0148	0.81 (0.69, 0.95)	0.0093

Model I is non-adjusted. Model II is adjusted for age and sex. Model III is adjusted for age, sex, ALT, AST, TC, TG, UA, and FBG.

### Ln-SII index is associated with the prevalence of adenomatous polyps in both sexes but not non-adenomatous polyps

Subgroup analyses based on key demographic characteristics were conducted to elucidate the relationship between the Ln-SII index and the prevalence of colorectal polyps, including both adenomatous and non-adenomatous types. As shown in Table 3, after adjusting for age, TG, TC, HDL-C, and LDL-C, the Ln-SII index exhibited an independent association with CPs and adenomatous polyps in both male and female participants. In Model I, the analysis revealed a significant association between the Ln-SII index and CPs in males (OR = 0.68, 95% CI: 0.57–0.80, *p* < 0.0001), while no significant association was found in females (OR = 1.03, 95% CI: 0.84–1.25, *p* = 0.7976). Specifically for adenomatous polyps, males demonstrated an OR of 0.69 (95% CI: 0.53–0.90, *p* = 0.0056), while females showed a non-significant association (OR = 0.78, 95% CI: 0.56–1.08, *p* = 0. 1,293). After adjusting for age in Model II, the association remained significant in males (OR = 0.69, 95% CI: 0.53–0.90, *p* = 0.0054), but non-significant in females (OR = 0.77, 95% CI: 0.56–1.08, *p* = 0. 1,275). In Model III, which accounted for additional confounding factors, the Ln-SII index maintained a robust association with adenomatous polyps in males (OR = 0.54, 95% CI: 0.39–0.76, *p* = 0.0003), whereas the association in females approached significance (OR = 0.71, 95% CI: 0.48–1.06, *p* = 0.0910). Conversely, no significant relationship was observed between the Ln-SII index and non-adenomatous polyps in either sex across all models, underscoring the specificity of the association with adenomatous polyps.

**Table 3:** Adjusted ORs and 95% CIs for the occurrence of colorectal polyps, including adenomatous and non-adenomatous polyps, in relation to the Ln-SII index, based on multinomial logistic regression with subgroup analyses by gender.

Variable	Model I		Model II		Model III	
	OR, 95% CI	*p*-value	OR, 95% CI	*p*-value	OR, 95% CI	*p*-value
Male						
Colorectal polyps						
Ln-SII	0.68 (0.57, 0.80)	<0.0001	0.67 (0.57, 0.80)	<0.0001	0.66 (0.54, 0.81)	<0.0001
Adenomatous polyps						
Ln-SII	0.69 (0.53, 0.90)	0.0056	0.69 (0.53, 0.90)	0.0054	0.54 (0.39, 0.76)	0.0003
Non-adenomatous polys						
Ln-SII	0.69 (0.58, 0.82)	<0.0001	0.68 (0.57, 0.81)	<0.0001	0.70 (0.57, 0.87)	0.0014
Female						
Colorectal polyps						
Ln-SII	1.03 (0.84, 1.25)	0.7976	1.05 (0.86, 1.27)	0.6398	0.91 (0.72, 1. 14)	0.4082
Adenomatous polyps						
Ln-SII	0.78 (0.56, 1.08)	0.1293	0.77 (0.56, 1.08)	0.1275	0.71 (0.48, 1.06)	0.0910
Non-adenomatous polys						
Ln-SII	1. 12 (0.91, 1.39)	0.2836	1. 15 (0.93, 1.42)	0.1981	0.99 (0.77, 1.28)	0.9561

Model I is non-adjusted. Model II is adjusted for age. Model III is adjusted for age, ALT, AST, TC, LDL-C, HDL-C, UA, and FBG.

### Ln-SII index shows inverted U-shaped relationship with colorectal polyp prevalence

**Figure 2:**
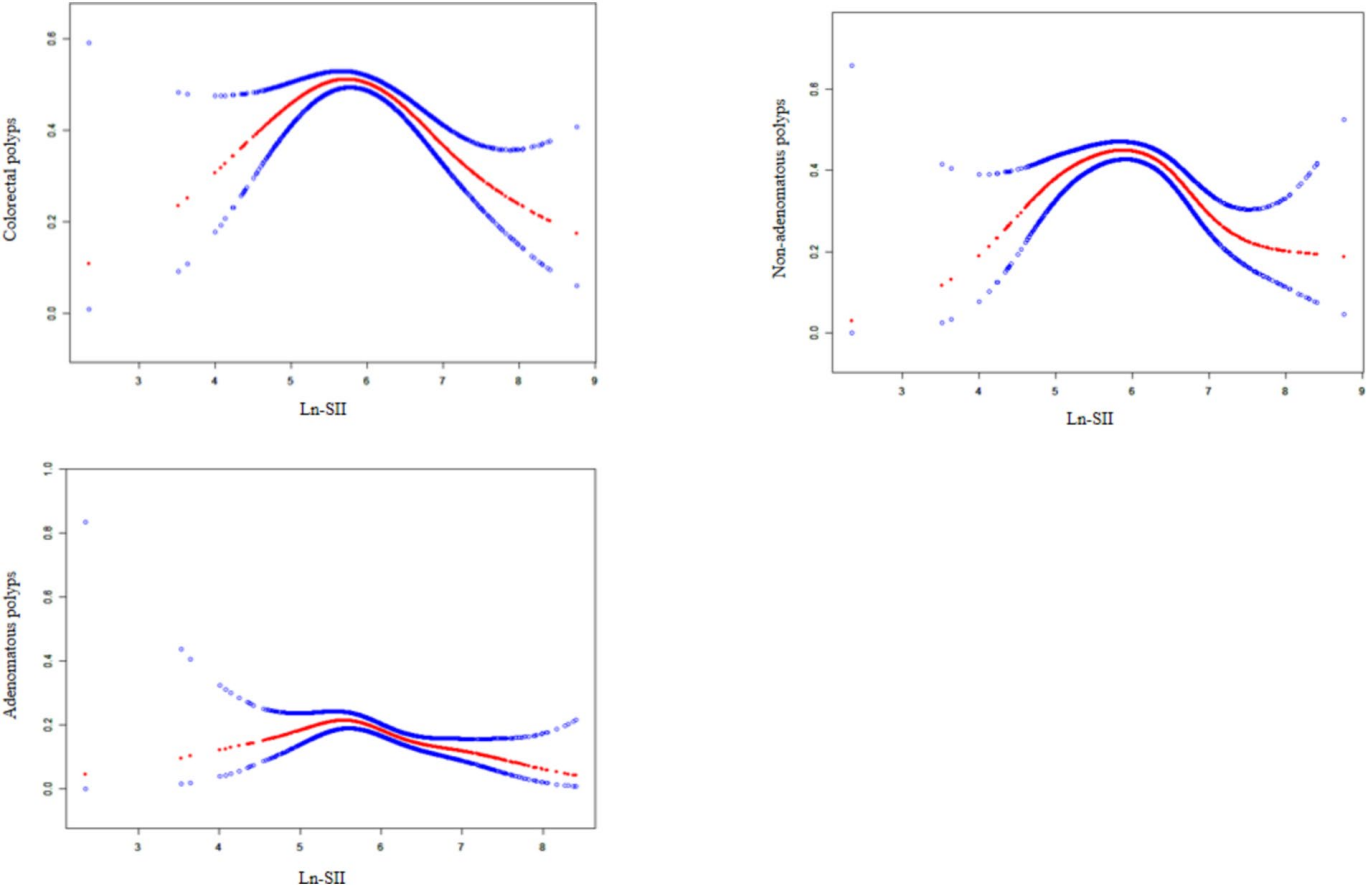
The association between Ln-SII and colorectal polyps. A solid rad line represents the smooth curve fit between variables. Blue bands represent the 95% of confidence interval from the fit.

As illustrated in Figure 2, smooth curve fitting analysis revealed an inverted U-shaped relationship between the Ln-SII index and the prevalence of CPs, including both adenomatous and non-adenomatous types. The saturation threshold effect analysis, as presented in Table 4, identified inflection points at 5.78 for overall CPs, 5.39 for adenomatous polyps, and 5.79 for non-adenomatous polyps. Before these thresholds, a significant positive correlation was observed, with an OR of 1.73 (95% CI: 1.25–2.40, *p* = 0.0009) for CPs. This association was more pronounced for adenomatous polyps, yielding an OR of 2.91 (95% CI: 1.03–8.20, *p* = 0.0437), while for non-adenomatous polyps, the OR was 1.86 (95% CI: 1.31–2.65, *p* = 0.0006). After surpassing the inflection point of 5.78, the association between the Ln-SII index and colorectal polyps remained significant, with an OR of 0.56 (95% CI: 0.46–0.68, *p* < 0.0001). This trend was consistent for both adenomatous (OR = 0.57, 95% CI: 0.43–0.74, *p* < 0.0001) and non-adenomatous polyps (OR = 0.57, 95% CI: 0.46–0.70, *p* < 0.0001).

**Table 4:** Threshold effect analysis of the Ln-SII index on the occurrence of colorectal polyps, including adenomatous and non-adenomatous polyps, using a two-piecewise linear regression model.

Colorectal polyps	OR, 95% CI	*p*-value
Ln-SII index		
Fitting by standard linear model	0.81 (0.72, 0.92)	0.0015
Fitting by two-piecewise linear model		
Inflection point	5.78	
<5.78	1.73 (1.25, 2.40)	0.0009
>5.78	0.56 (0.46, 0.68)	<0.0001
Log-likelihood ratio	<0.001	
Adenomatous polyps	OR, 95% CI	P value
Ln-SII index		
Fitting by standard linear model	0.72 (0.59, 0.89)	0.0018
Fitting by two-piecewise linear model		
Inflection point	5.39	
<5.39	2.91 (1.03, 8.20)	0.0437
>5.39	0.57 (0.43, 0.74)	<0.0001
Log-likelihood ratio	0.001	
Non-adenomatous polyps	OR, 95% CI	P value
Ln-SII index		
Fitting by standard linear model	0.84 (0.74, 0.97)	0.0148
Fitting by two-piecewise linear model		
Inflection point	5.79	
<5.79	1.86 (1.31, 2.65)	0.0006
>5.79	0.57 (0.46, 0.70)	<0.0001
Log-likelihood ratio	<0.001	

Age, sex were adjusted.

## Discussion

CPs are highly prevalent worldwide, with detection rates ranging from 10 to 20% during colonoscopic screening. The incidence of CPs is notably higher in males than in females, which may be attributed to the strong correlation between CPs and colorectal cancer (CRC) (11, 12). Intestinal polyps arise from an imbalance between the proliferation and apoptosis of colorectal epithelial cells, leading to abnormal mucosal hyperplasia (13). As reported by Strum (14), the majority of CRC cases develop from the malignant transformation of conventional adenomas and sessile serrated polyps, underscoring adenomas as key precursors of CRC and emphasizing the increased cancer risk associated with their presence. Early detection and removal of CPs have been shown to significantly reduce both the incidence and mortality of CRC, as demonstrated by Helsingen and Kalager (15). Various screening modalities, including fecal immunochemical tests (FIT) and colonoscopy, are routinely employed in clinical practice. However, the implementation of screening programs faces several challenges, such as limited accessibility, the need for individualized screening strategies, and the influence of health disparities on screening effectiveness and patient compliance. Moreover, the precise pathogenesis of CPs remains incompletely understood.

In this cross-sectional study involving 3,028 Chinese patients, we performed logistic regression analysis and found a significant association between the ln-SII and the prevalence of CPs, particularly adenomatous polyps. This association was more pronounced in males. Further analysis using smooth curve fitting revealed a non-linear, inverted U-shaped relationship between ln-SII and the prevalence of CPs. This association was evident in both adenomatous and non-adenomatous polyps, with clear inflection points at ln-SII values of 5.39 and 5.79, respectively. Below these thresholds, higher ln-SII levels were significantly associated with an increased risk of CP. In contrast, above these inflection points, higher ln-SII levels were associated with a decreased risk of polyp formation.

Extensive studies have demonstrated a robust link between systemic inflammatory markers and increased cancer risk and mortality (16–18). Chronic inflammation has been firmly established as a driving factor in the pathogenesis of CRC (19). For example, a large-scale cohort study revealed that genetic factors influencing intestinal barrier function and inflammatory responses increase susceptibility to CRC, particularly in European populations. This supports the hypothesis that genetic predispositions, altered intestinal permeability, and microbial endotoxemia contribute to CRC progression (20). These results are consistent with our findings, which demonstrate an association between the SII and the prevalence of CPs. However, research exploring pre-diagnostic associations between systemic inflammation and cancer risk remains limited (21).

Neutrophils, or polymorphonuclear leukocytes, are pivotal in the innate immune response and exhibit several pro-tumor characteristics. They facilitate cancer progression through mechanisms such as promoting angiogenesis, inducing immune suppression, and aiding metastasis (22). By releasing cytokines and growth factors, neutrophils contribute to an inflammatory microenvironment that fosters tumor cell growth and spread (23). Furthermore, the interactions between platelets and tumor cells—both direct and indirect—help sustain tumor growth, enhance metastasis, support immune evasion, and contribute to chemoresistance (24). In contrast, lymphocytes are essential effector cells in anti-cancer immunity. Higher proportions of peripheral lymphocytes prior to treatment have been associated with improved prognosis in several cancers, including CRC (25).

The systemic immune inflammation index (SII), which integrates markers of both pro-inflammatory and anti-inflammatory cells, offers a comprehensive reflection of the immune landscape. In the context of CRC, the balance between neutrophils, platelets, and lymphocytes plays a crucial role in tumor progression (16). Elevated neutrophil and platelet counts, indicative of a pro-inflammatory state, are often associated with poor prognosis, while higher lymphocyte counts reflect a stronger anti-tumor immune response (26). Our findings suggest an inverted U-shaped association between SII and the risk of CPs, indicating that moderate systemic inflammation may foster an environment conducive to polyp growth. Specifically, low SII levels may reflect insufficient immune activation, failing to stimulate the inflammatory processes necessary for initiating polyp growth. Conversely, excessively high SII levels correspond to a chronic inflammatory state that could exceed the optimal threshold, leading to immune exhaustion or tissue damage, which may limit further lesion development. Moderate levels of inflammation appear to provide an “activation zone” that promotes the growth and maintenance of CPs, highlighting the complex role of systemic inflammation.

The clinical importance of the inverted U-shaped association lies in its ability to illuminate the dual role of systemic inflammation. Unlike linear relationships, which suggest that either increasing or decreasing inflammation would uniformly alter risk, the U-shaped relationship emphasizes that both insufficient and excessive inflammation may be detrimental in different ways. Therapeutically, this underscores the need to avoid suppressing inflammation too strongly or allowing unchecked chronic inflammation. From a statistical perspective, the U-shaped model better captures the non-linear and dynamic relationship between SII and colorectal polyp development compared to linear or monotonic models. Linear models oversimplify the interaction between inflammation and polyp formation, potentially leading to inaccurate conclusions or ineffective interventions. The U-shaped relationship provides a more realistic and nuanced framework, enabling refined risk stratification and personalized therapeutic strategies that consider the complexity of immune-inflammatory interactions.

Further studies are necessary to explore the molecular mechanisms underlying the inverted U-shaped association observed in this study. The interplay between systemic inflammation, immune cell activation, and tumor microenvironment dynamics remains a critical area for future research. Understanding these complex interactions could refine risk stratification for CRC and inform the development of personalized strategies for early detection and prevention, taking into account individual immune-inflammatory profiles.

In comparison to previous research, our study offers novel insights into the relationship between SII and CPs. Chen et al. (7), in a retrospective analysis of 1,383 CRC patients post-radical surgery, demonstrated that SII is a robust prognostic tool for predicting survival outcomes in CRC patients. Their findings suggest that SII may aid in identifying high-risk patients, even within the same TNM stage. Similarly, Menyhart et al. (27), in a meta-analysis of both prospective and retrospective studies, concluded that both the SII and the systemic inflammation response index (SIRI) are significantly associated with poorer overall survival (OS) and disease-free survival (DFS/RFS) in CRC patients, indicating their potential utility as prognostic biomarkers. Additionally, Feng et al. (28) conducted a retrospective study involving patients newly diagnosed with CRC or colorectal adenomatous polyps. Among 342 patients (216 with CRC and 126 with adenomatous polyps), their findings indicated that inflammation-related markers—such as lymphocyte count, monocyte count, and mean platelet volume—may serve as potential indicators for early CRC diagnosis. Notably, we identified an inverted U-shaped relationship between ln-SII and CP prevalence, suggesting that both low and high levels of systemic inflammation may contribute to polyp formation through distinct biological mechanisms, contrasting with the linear associations reported in prior studies.

The observed inverted U-shaped association between ln-SII and CP risk may reflect the dual role of inflammation in carcinogenesis. At lower SII levels, moderate inflammation may promote epithelial cell proliferation, thereby facilitating polyp initiation and growth. However, once a critical threshold of inflammation is exceeded, heightened systemic inflammation may create a microenvironment that inhibits polyp formation, potentially through immune-mediated cytotoxic mechanisms that suppress neoplastic growth. This hypothesis aligns with emerging evidence on the complex role of the immune system in tumorigenesis and cancer progression (26).

The identification of this inverted U-shaped relationship between SII and CP risk suggests the potential utility of SII as a biomarker for assessing polyp risk in clinical practice. Monitoring fluctuations in SII could aid in the early detection of individuals at increased risk for polyp development, enabling timely intervention and surveillance. As proposed by Zhu et al. (29), the relationship between CRP levels and the risk of colorectal cancer is nonlinear, exhibiting a “fast-to-low increase” pattern. Given the well-established progression from adenomatous polyps to CRC, incorporating SII into routine risk assessment protocols has the potential to refine the predictive accuracy of CRC screening and strengthen the effectiveness of preventive strategies.

Nevertheless, the cross-sectional design of our study limits our ability to infer causal relationships between SII and CP development. Although we accounted for multiple confounders, residual confounding cannot be entirely excluded. This is particularly relevant for lifestyle factors such as patients’ dietary habits, including alcohol consumption, which were not systematically assessed in this study. Additionally, our study cohort consisted exclusively of Chinese patients, potentially limiting the generalizability of our findings to other ethnic groups. To confirm these associations and elucidate the underlying biological mechanisms, future longitudinal studies involving multi-ethnic cohorts are warranted. Prospective research is needed to validate the inverted U-shaped relationship between SII and CP risk across diverse populations and to further investigate the biological pathways involved. Furthermore, evaluating the integration of SII into current risk prediction models may enhance their predictive capacity and utility in CRC screening programs.

## Conclusion

In conclusion, our study identifies an inverted U-shaped association between SII and the risk of CPs, suggesting that variations in SII may have clinical relevance. While these findings indicate that SII could potentially be a useful indicator in the context of CP development, further investigations are needed, particularly involving comparisons across different polyp grades and evaluations of specificity, sensitivity, and accuracy. Such studies would be crucial to fully assess the predictive value of SII in CRC prevention efforts.
